# Pilot Testing of the UB-CAM Delirium Screening App

**DOI:** 10.1093/geroni/igab046.3515

**Published:** 2021-12-17

**Authors:** Ashley Kuzmik, John Joseph Hannan, Long Ngo, Marie Boltz, Priyanka Shrestha, Sharon Inouye, Donna Fick, Edward Marcantonio

**Affiliations:** 1 Penn State University, University Park, Pennsylvania, United States; 2 Pennsylvania State University, State College, Pennsylvania, United States; 3 Harvard T.H. Chan School of Public Health, Brookline, Massachusetts, United States; 4 Pennsylvania State University, University Park, Pennsylvania, United States; 5 Betty Irene Moore School of Nursing (UC-DAVIS Health); 6 Harvard Medical School, Boston, Massachusetts, United States; 7 Beth Israel Deaconess Medical Center, Boston, Massachusetts, United States

## Abstract

Systematic screening improves delirium detection among hospitalized older adults. This poster describes the development and pilot testing of an iOS-based app that incorporates the Ultra-Brief Confusion Assessment Method (UB-CAM), a two-step, delirium detection protocol that combines the UB-2 (2-item screener) and 3D-CAM. Previous work tested a RedCAP-based UB-CAM app in 527 patients with 399 physicians, nurses, and certified nursing assistants (CNAs) showing it can be successfully completed by all three disciplines in 97% of eligible patients in 80 seconds on average with over 85% accuracy relative to a gold standard. To improve accessibility to the clinical setting, our research team now collaborated with a computer scientist to develop and refine an iOS-based UB-CAM app for the iPhone and iPad through iterative “laboratory” testing. The app was piloted by non-clinician, research testers in hospitalized older adults (age x̄ =83, SD= 8.0) with dementia (Clinical Dementia Rating Scale x̄ =1.1, SD= .30); 64% were assessed to be delirium positive. The app demonstrated preliminary efficiency (90 seconds on average), high acceptability (100% satisfaction of users), and reliability (100% inter-rater). This project underscores the need for close collaboration between researchers, clinicians, and computer scientists with iterative testing of bedside-facing apps prior to testing with patients. Next steps include testing effectiveness in a pragmatic trial with clinician users (physicians, nurses, CNAs), integrating the UB-CAM app into the routine hospital care of all older patients. Having rapid, accurate bedside delirium detection has the potential to transform care.

